# Adsorption Studies of Volatile Organic Compound (Naphthalene) from Aqueous Effluents: Chemical Activation Process Using Weak Lewis Acid, Equilibrium Kinetics and Isotherm Modelling

**DOI:** 10.3390/ijms22042090

**Published:** 2021-02-19

**Authors:** Adeola A. Akinpelu, Zaira Zaman Chowdhury, Shahjalal Mohd. Shibly, Abu Nasser Mohd Faisal, Irfan Anjum Badruddin, Md. Mahfujur Rahman, Md. Al Amin, Suresh Sagadevan, Omid Akbarzadeh, T. M. Yunus Khan, Sarfaraz Kamangar, Khalisanni Khalid, R. Saidur, Mohd Rafie Johan

**Affiliations:** 1 Nanotechnology and Catalysis Research Center, University of Malaya, Kuala Lumpur 50603, Malaysia or dr.adeolahakeem799@gmail.com (A.A.A.); shiblygsa@gmail.com (S.M.S.); faisal_dhaka2003@yahoo.com (A.N.M.F.); sureshsagadevan@gmail.com (S.S.); omid@um.edu.my (O.A.); mrafiej@um.edu.my (M.R.J.); 2 Center of Environment and Water, King Fahd University of Petroleum and Minerals, Dhahran 34464, Saudi Arabia; 3 Department of Mechanical Engineering, College of Engineering, King Khalid University, P.O. Box 394, Abha 61421, Saudi Arabia; magami.irfan@gmail.com (I.A.B.); yunus.tatagar@gmail.com (T.M.Y.K.); sarfaraz.kamangar@gmail.com (S.K.); 4 Research Center of Advanced Materials and Science (RCAMS), King Khalid University, P.O. Box 9004, Abha 61413, Asir, Saudi Arabia; 5 Islamic Business School, University Utara Malaysia, Kedah 06010, Malaysia; dr_mahfuj@yahoo.co.uk; 6 Global Centre for Environmental Remediation (GCER), University of Newcastle, Callaghan, NSW 2308, Australia; M.AlAmin@uon.au; 7 Malaysian Agricultural Research & Development Institute (MARDI), Serdang 43000, Malaysia; sanni@mardi.gov.my; 8 Biocomposite Technology Laboratory, Institute of Tropical Forestry and Forest Product (INTROP), University Putra Malaysia (UPM), Serdang 43400, Selangor, Malaysia; 9 Research Centre for Nano-Materials and Energy Technology (RCNMET), School of Science and Technology, Sunway University, No. 5, Jalan Universiti, Bandar Sunway, Petaling Jaya 47500, Malaysia; prof.saidur@gmail.com

**Keywords:** chemical activation process, weak Lewis acid, activated carbon, date seeds (DS), water purification, volatile organic compound (napththalene-C_10_H_8_)

## Abstract

This study deals with the preparation of activated carbon (CDSP) from date seed powder (DSP) by chemical activation to eliminate polyaromatic hydrocarbon—PAHs (naphthalene—C_10_H_8_) from synthetic wastewater. The chemical activation process was carried out using a weak Lewis acid of zinc acetate dihydrate salt (Zn(CH_3_CO_2_)_2_·2H_2_O). The equilibrium isotherm and kinetics analysis was carried out using DSP and CDSP samples, and their performances were compared for the removal of a volatile organic compound—naphthalene (C_10_H_8_)—from synthetic aqueous effluents or wastewater. The equilibrium isotherm data was analyzed using the linear regression model of the Langmuir, Freundlich and Temkin equations. The R^2^ values for the Langmuir isotherm were 0.93 and 0.99 for naphthalene (C_10_H_8_) adsorption using DSP and CDSP, respectively. CDSP showed a higher equilibrium sorption capacity (*q_e_*) of 379.64 µg/g. DSP had an equilibrium sorption capacity of 369.06 µg/g for C_10_H_8_. The rate of reaction was estimated for C_10_H_8_ adsorption using a pseudo-first order, pseudo-second order and Elovich kinetic equation. The reaction mechanism for both the sorbents (CDSP and DSP) was studied using the intraparticle diffusion model. The equilibrium data was well-fitted with the pseudo-second order kinetics model showing the chemisorption nature of the equilibrium system. CDSP showed a higher sorption performance than DSP due to its higher BET surface area and carbon content. Physiochemical characterizations of the DSP and CDSP samples were carried out using the BET surface area analysis, Fourier-scanning microscopic analysis (FSEM), energy-dispersive X-ray (EDX) analysis and Fourier-transform spectroscopic analysis (FTIR). A thermogravimetric and ultimate analysis was also carried out to determine the carbon content in both the sorbents (DSP and CDSP) here. This study confirms the potential of DSP and CDSP to remove C_10_H_8_ from lab-scale synthetic wastewater.

## 1. Introduction

Polycyclic aromatic hydrocarbons (PAHs) are considered highly toxic, as well as persistent, environmental contaminants. This group of compounds contains multiple groups of aromatic rings infused together, which have abilities to metabolize and yield some harmful derivatives [1,2]. Polycyclic aromatic hydrocarbons (PAHs) are volatile organic compounds. Consequently, these derivatives, when released into the environment without careful monitoring, can react with DNA to stimulate carcinogenic and mutagenic responses in living organisms [1,2]. Due to their persistent nature, high toxicity and easy mobility, the European Environmental Protection (EEA) Agency, as well as the World Health Organization, classified it as a priority pollutant that needs to be monitored strictly in any industrial effluents and recommended a safe, tolerable limit in water for public consumption [3,4]. In spite of these strict regulations, a lot of relevant studies have illustrated that the inadequate combustion of fossil fuels (mainly coke or coal) with the incineration of waste are the two most leading sources for emissions of PAHs in the air, soil and overall aquatic environment [5,6]. It was observed that around 20–30% of PAHs are emitted from power plants burning coal for energy [7,8]. It was estimated that coal burning per ton can emit 1.56 mg of PAHs into the environment, which is quite alarming [7,8]. Furnaces used for pulverizing coal can generate 8.8–16.3 μg·m^3^ [9]. The application of activated carbon with requisite properties for the adsorption of PAHs from aqueous effluents is considered one of the most feasible techniques for its cost effectiveness, simple operational features with regeneration efficiencies [3,10]. In this research, naphthalene (C_10_H_8_) was chosen as an adsorbate that is a representative of PAHs and is widely found in waste aqueous stream due to careless industrial discharge.

The imminent shortage of nonrenewable fossil fuels has been levied to search substitute materials to produce nano and microporous carbons suitable for the adsorption of persistent organic, especially nonpolar, volatile aromatic compounds from waste streams. Lignocellulosic biomass residues (LSM) are ecofriendly, renewable—thus, cost-effective—sustainable, greener resources to generate high-quality carbon, dependent on the appropriate selection of process parameters for production [11,12,13,14]. Hence, LSM residues have been widely used to produce activated carbon for environmental remediation for the last few decades. However, the complete mechanism of volatile aromatic compound (PAH) adsorption onto the surface of the lignocellulosic substrate (LSM) itself or the activated carbon derived from it is, to some extent, yet uncertain [3,11]. Inspite of the growing trend for exploiting LSM residues to yield valuable solid, liquid and gaseous chemicals, this impending resource is sometimes not utilized properly or even dumped as solid waste to decompose and burnt in certain developing countries [10]. The thermochemical conversion or pyrolysis of LSM wastes can yield activated carbon with effective physiochemical properties for versatile applications [11]. To date, a lot of research has been carried out to produce carbon adsorbent from LSM substrates. Earlier, date stone was activated to obtain activated carbon by the steam activation technique where the surface area of the prepared carbon reached up to 635 m^2^/g [12]. Some research has demonstrated that the activation process using one-step CO_2_ gas can produce carbon with micropores, while, in two-step activation processes, using CO_2_ can produce carbon having a mixture of micro and mesopores [10,13,14,15,16].

To date, different types of LSM waste residues, such as corn cob [17], apricot stone [18,19], date pits [20], peach stone [21], corn stover [22], coconut shell [23], grape seed [24], cherry stone [25], rice husk [26], etc., have been converted to carbon, having suitable porous textures. However, the application of different types of chemicals as an activating agent can significantly change the overall qualities of carbon [27,28]. The organic complex present in LSM waste can produce tar, which can cause blockage of the pores during the activation process. The application of acidic or basic types of agents can promote pore formations, leading to enlarged surface areas. This will help in dehydration reactions and initiate the burning of tars to yield carbon with desirable properties [29,30]. PAHs have high molecular weights with larger size molecular dimensions [31]. Therefore, it can be easily understood that activated carbon with an adequate porous texture would be useful for the adsorptive removal of PAHs from an aqueous stream. LSM precursors having high cellulosic contents usually give activated carbon with a microporous texture, which is suitable for the adsorption of gaseous molecules, as well as heavy metals, having smaller ionic sizes. However, the starting materials having higher lignin contents will produce activated carbon containing mesopores inside them [32,33,34]. Thus, process variables like the pyrolysis temperature, gas flow rate, time, ratio and chemical activation agent for impregnation should be carefully monitored. In this research, preliminary studies were performed to optimize the temperature, time and impregnation ratio using zinc acetate to ensure the maximum adsorption uptake of the adsorbate naphthalene (C_10_H_8_) molecules. In this research, date stone-based activated carbon was prepared using zinc acetate as an activating agent, which is comparatively less harsh, a weak Lewis acid, than other inorganic strong Lewis acids of H_3_PO_4_, H_2_SO_4_ or concentrated Lewis base solutions like KOH or NaOH. The ratio of date seed powder with zinc acetate salt was kept to a minimum at 2:1 to avoid corrosion inside the pyrolysis equipment. Batch adsorption for the removal of naphthalene (C_10_H_8_) was carried out using both LSM wastes of date seed powder (DSP) and its activated carbon (CDSP). The reaction kinetics in terms of the pseudo-first and second orders were evaluated. The mechanism of adsorption was determined using the intraparticle diffusion model. The equilibrium isotherm model parameters were determined. The physiochemical characterizations of both the DSP and CDSP samples were carried out in terms of a Fourier-transform emission scanning electron microscope (FESEM), ultimate analysis, BET surface area and FTIR analysis. The findings of this study showed that weak Lewis acid-based chemical activation using zinc acetate dehydrate salt (Zn(CH_3_CO_2_)_2_·2H_2_O]) is suitable to develop porous carbon, which can entrap naphthalene (C_10_H_8_) molecules from wastewater.

## 2. Results and Discussions

### 2.1. Effect of Contact Time on the Adsorption Capacity of Naphthalene (C_10_H_8_)

**Figure 1:**
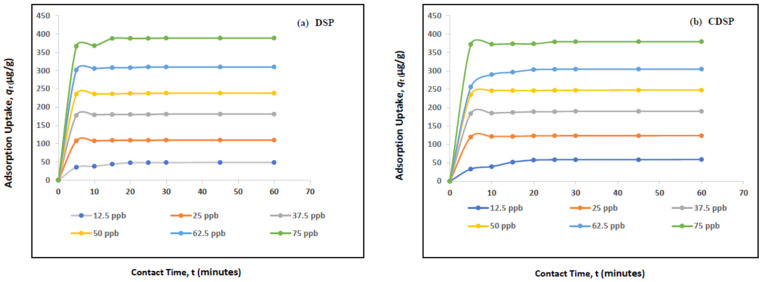
Effect of the contact time (minutes) on the adsorption uptake, *q_t_* (μg/g), of naphthalene (C_10_H_8_) onto (**a**) date seed powder (DSP) and (**b**) carbon date seed powder (CDSP) (concentration 12.5 ppb–75 ppb, pH = 6.5, temperature = 30 °C and agitation speed = 200 rpm).

Figure 1 illustrates the effect of the contact time on the removal process of naphthalene (C_10_H_8_) using DSP and CDSP, where the initial concentration of naphthalene was changed from 12.5 ppb to 75 ppb. The system reached equilibrium within 30 min. Each five-min interval, the residual concentration of naphthalene was measured. The activated sample of CDSP showed more sorption capacity than the unactivated, raw DSP sample, which was expected due to the enlarged surface area and well-developed pore size distribution of CDSP, which will be described in the characterization Section 2.4.1. The adsorption process can be evidently divided into two main stages. The initial stage was very fast, and it was for the first 5 min, where the adsorption rate was faster. It seems the maximum of the naphthalene molecules (C_10_H_8_) were adsorbed for first five min. The second stage was slow, and the curve became almost parallel to the *x*-axis, reflecting a negligible change in the sorption amount. Within 30 min, it became constant, i.e., the system reached equilibrium.

### 2.2. Adsorption Isotherms

The surface properties, as well as affinity, of the adsorbent materials towards the adsorbate can be predicted by analyzing the equilibrium data using different isotherm models, mainly Langmuir, Freundlich and Temkin isotherm models [35,36].

The model parameters are evaluated and summarized in Table 1.

In this work, the Langmuir isotherm showed better R^2^ values than the other two models. This suggests that the adsorption of C_10_H_8_ onto DSP and CDP was a monolayer, and the homogenous interaction dominated the adsorption process [10,35,36,37]. The magnitude of n obtained for the Freundlich model was greater than 1, reflecting the strong affinity between C_10_H_8_ with DSP and CDSP samples both (Table 1).

### 2.3. Adsorption Kinetics Study

**Figure 2:**
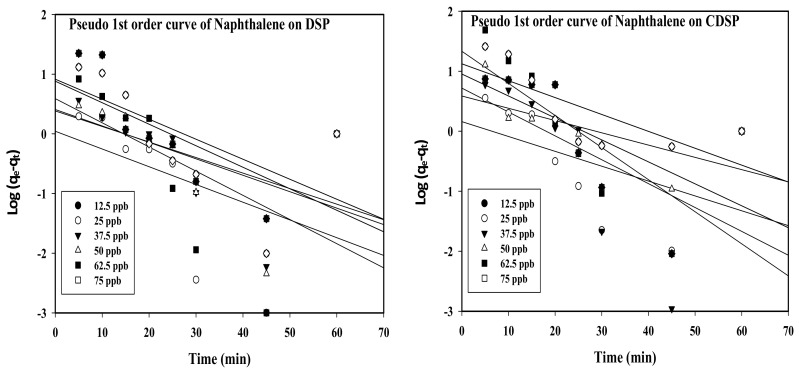
Linear regression plots for pseudo-first order kinetics of naphthalene (C_10_H_8_) adsorption onto DSP and CDSP (concentration 12.5 ppb–75 ppb, pH = 6.5, temperature = 30 °C and agitation speed = 200 rpm).

**Figure 3:**
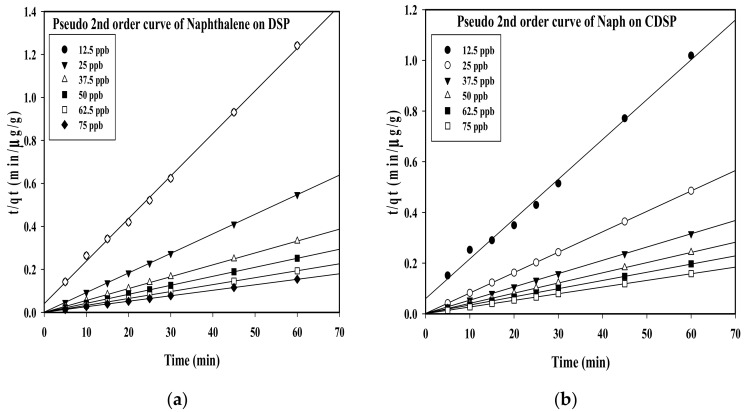
Linear regression plots for pseudo-second order kinetics of naphthalene (C_10_H_8_) adsorption onto (**a**) DSP and (**b**) CDSP (concentration 12.5 ppb–75 ppb, pH = 6.5, temperature = 30 °C and agitation speed = 200 rpm).

Adsorption kinetics can provide information regarding the rate of reaction at a constant pressure or temperature [38,39]. Linear plots for pseudo-first and second order kinetics for naphthalene (C_10_H_8_) adsorption over DSP and CDSP are shown in Figure 2 and Figure 3.

**Figure 4:**
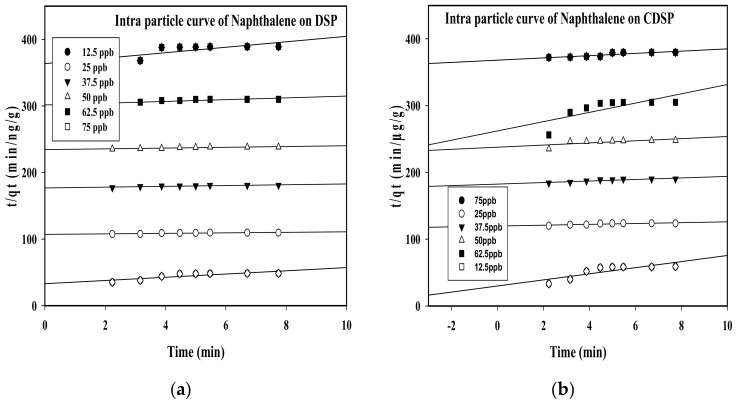
Linear regression plots for the intraparticle diffusion of naphthalene (C_10_H_8_) adsorption onto (**a**) DSP and (**b**) CDSP (concentration 12.5 ppb–75 ppb, pH = 6, temperature = 30 °C and agitation speed = 200 rpm).

Figure 2, representing linear regression plots for pseudo-first order kinetics, showed scattered data points for naphthalene (C_10_H_8_) adsorption onto DSP and CDSP. This implied that the present equilibrium system did not follow the pseudo-first order kinetic model, which represents the physical nature of the adsorption process. However, the data points are nicely arranged beside the linear plots obtained for the pseudo-second order kinetic model. This showed the chemical nature of the adsorption process (Figure 3). The linear plots obtained for the intraparticle diffusion process did not cross the origin (Figure 4). This reflected that the rate-controlling steps for the adsorption process were not only controlled by pore diffusion, some other mechanisms of dipole-dipole interactions and hydrogen bonding took place during the adsorption process [10,40,41]. From the linear plots (Figure 2, Figure 3 and Figure 4), the pseudo-first and second and intraparticle diffusion plot variables and constants are estimated and summarized in the following Table 2 and Table 3.

It is observed that the linear regression coefficient, *R*^2^, obtained for the pseudo-second order model showed better values near to unity compared to the pseudo-first order model for naphthalene (C_10_H_8_) adsorption onto the CDSP sample (Table 3). Furthermore, the experimental *q_e_* obtained for this model was nearly equivalent with the calculated *q_e_* values after linearization (Table 3). This reflected that, over the CDSP sample, the adsorption process of C_10_H_8_ proceeded via chemical interactions mainly [10,37,38,39]. On the contrary, C_10_H_8_ adsorption onto the unactivated, raw DSP sample by the pseudo-first order model also showed better linearity, except concentrations 25 and 37.5 ppb (Table 2). Over the DSP sample, all the concentration ranges (12.5–75 ppb) under investigation followed the pseudo-second order model (Table 2). This implied, over the DSP sample, C_10_H_8_ adsorption also proceeded via the chemical adsorption process [10,40,41]. As the concentration range increased, both the experimental and calculated *q_e_* values increased. Similarly, the boundary layer effect, *C_i_*, observed for the adsorption onto DSP and CDSP also showed an increasing trend (Table 2 and Table 3) with the concentration [40,41].

**Figure 5:**
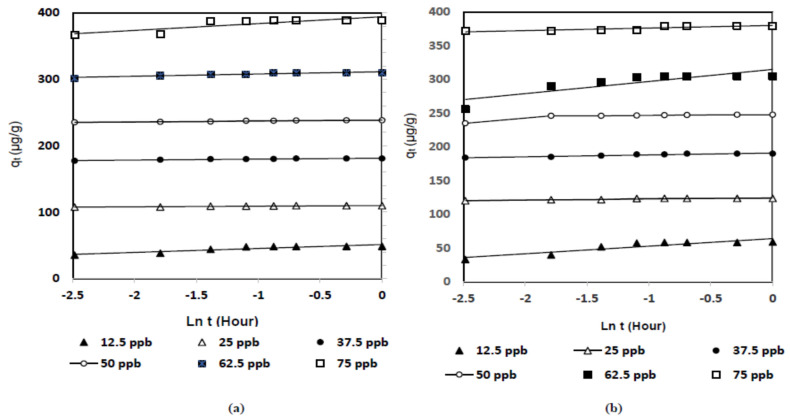
Linear regression plots for the Elovich model for naphthalene (C_10_H_8_) adsorption onto (**a**) DSP and (**b**) CDSP (concentration 12.5 ppb–75 ppb, pH = 6, temperature = 30 °C and agitation speed = 200 rpm).

The experimental data were further analyzed using the Elovich kinetic model. The linear plots for the Elovich model used for naphthalene (C_10_H_8_) adsorption onto the DSP and CDSP samples are illustrated in Figure 5.

From Table 4, it is observed that the *R*^2^ values obtained for the Elovich equation for naphthalene (C_10_H_8_) sorption onto the DSP and CDSP samples were better than the pseudo-first order model. This showed the chemisorption nature of the adsorption process. The values for the model constant 1/b *Ln* (ab) increased subsequently with the increasing concentration under investigation. This increasing trend was expected. With the increasing concentration, more naphthalene (C_10_H_8_) molecules can successfully collide with the adsorbents’ active sites, resulting in greater removal efficiencies [10].

### 2.4. Physical Characterizations of the Adsorbent (DSP and CDSP)

#### 2.4.1. Surface Area Analysis

In the case of LSM materials like DSP, cellulose and hemicellulose are embedded inside the lignin molecules. Thus, the structure becomes compact without a negligible amount of pores inside it. This results in a lower surface area with less volume of pores [42,43,44,45]. During the initial period of pyrolysis, at a relatively lower temperature range from 200 to 400 °C, the carbonization of polysaccharides (cellulose and hemicellulose) takes place, with the partial disintegration of lignin. However, at higher temperatures, starting from 400 to 1000 °C, lignin starts to decompose to form different types of volatile organic compounds and tar residues. Later on, with the subsequent increase of temperature, volatile compounds start to evaporate and leave as gaseous products from the pyrolysis unit [46]. This would considerably increase the surface area and pore volume. The tarry deposition blocking the pore would be either converted to carbon or leave as a volatile compound due to the application of a suitable activating agent under the optimized temperature and time of the pyrolysis process. The BET surface area, *t*-plot external surface area, *t*-plot micropore volume, micro and mesopore volume and diameter of the pore for the DSP and CDSP samples are listed in Table 5. In this work, after chemical pyrolysis using zinc acetate salt (Zn(CH_3_CO_2_)_2_·2H_2_O) at 1000 °C for 1 h, the surface area was almost enhanced by 99.43%. It was drastically enhanced from 7.30 m^2^/g to 725.85 m^2^/g, which is desirable for a better adsorption of naphthalene molecules (C_10_H_8_) from an aqueous stream. Before activation, the raw DSP sample was mainly microporous, having a pore diameter of 1.02 nm. After activation, using weak Lewis acids like Zn(CH_3_CO_2_)_2_·2H_2_O, the internal walls between the micropores were disintegrated to form larger-diameter mesopores having an internal diameter of 3.96 nm [47]. Mesopores are suitable for entrapping larger organic molecules during the adsorption process. The results illustrated that chemical activation using zinc acetate at 1000 °C for 1 h is suitable for the adsorption of naphthalene molecules from water.

**Figure 6:**
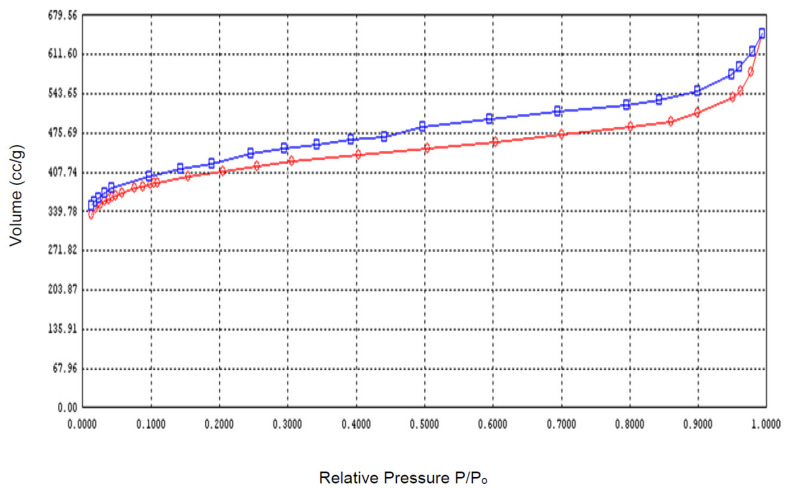
Nitrogen adsorption/desorption isotherm for the CDSP sample.

The macropore and mesopore and micropore volumes were determined from the mercury intrusion porosimeter. The macropore and mesopore volumes for the CDSP sample determined were 0.221 cm^3^/g and 0.4253 cm^3^/g. Thus, it showed that the macropore had a negligible contribution for the porous structure of the CDSP sample. A similar phenomenon has been reported about activated carbon obtained from apricot stone at different temperatures [48]. Following, Figure 6 shows the BET isotherm curve for the CDSP sample, which illustrates the presence of the micropores and mesopores inside the CDSP sample. Based on the IUPAC classification, the isotherm obtained for the CDSP sample can be categorized as a type II isotherm. After activation at a higher temperature in the presence of Zn(CH_3_CO_2_)_2_·2H_2_O, the surface area of the raw date stone increased drastically to 725.83 m^2^/gm. The wide microporous texture can be confirmed from the round knee shape of the curve at a relatively lower pressure. At a relative pressure of 0.9, the curve showed a slightly upward trend, indicating the presence of micropores, along with a certain amount of mesopores, inside the sample [49].

#### 2.4.2. Ultimate Analysis of DSP and CDSP

The ultimate analysis of raw DSP and its activated carbon CDSP sample is listed in Table 6. After chemical pyrolysis, the proportion of carbon increased in the CDSP sample. However, the hydrogen and oxygen contents reduced due to the pyrolysis process. This happened due to the disruption of weaker bonds during the pyrolysis process [50]. The H/C and O/C ratios showed a decreasing trend after the chemical activation due to the decarboxylation and dehydration reactions [51].

#### 2.4.3. Surface Morphological Analysis (DSP and CDSP)

**Figure 7:**
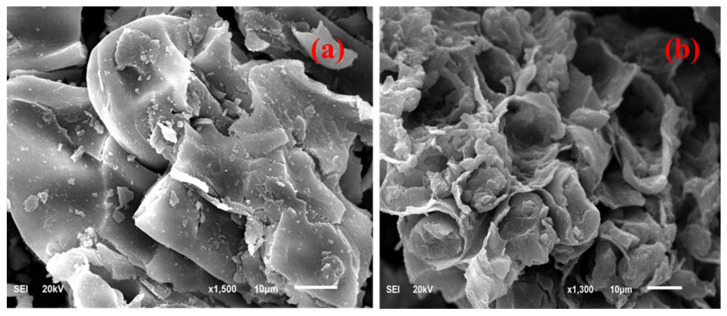
Surface morphological feature analysis using Fourier-transform emission scanning electron microscope (FESEM) images: (**a**) DSP and (**b**) CDSP.

Figure 7a,b illustrates the FESEM images of raw DSP and CDSP samples after the zinc acetate-based chemical pyrolysis process at 1000 °C after 60 min. Raw DSP showed a smooth texture without any pores. Some occasional flakes were deposited onto its surface, but the surface was even, without any tunnels or cracks (Figure 7a). Lignocellulosic waste (LSM) like raw DSP contains lignin, cellulose, hemicellulose, etc. as the major components, with some minor components like pectin and wax. Lignin and hemicellulose usually shield the cellulosic fiber in LSM wastes, making its surface relatively even free from folding, tunnels or cracks over its surface. A similar observation for FESEM images of raw *Durio zibethenus* (Durian wood) sawdust before carbon conversion was reported earlier [42]. However, after chemical activation using zinc acetate—which is acting here as a weak Lewis acid—the surface morphology and texture were changed substantially (Figure 7b). Basically, the constituents’ biopolymers of cellulose and hemicellulose, as well as lignin, in the DSP sample during the chemical pyrolysis process started to degrade, resulting in char and some tarry products at a relatively lower temperature. After that, with a subsequent increase of temperature until 1000 °C for 60 min, moisture and all types of volatile organic compounds, including the tarry residues, also burnt away and evaporated as a waste gas, leaving some irregular folds over the surface of the activated carbon (CDSP). The structure became porous, and the pores were also irregular in size and shape (Figure 7b). Thus, the volume of the micro and mesopores were enhanced, leading to a higher surface area of CDSP compared to DSP. Similar results for pore development over the carbonaceous char was observed earlier after dry torrefaction, i.e., carbonization at a lower temperature of 250 °C using *Adansonia digitata* (Baobab) tree trunk powder without the addition of any chemicals [43]. This phenomenon is further supported by our surface area analysis results summarized in Table 5 earlier. It was earlier reported also that strong Lewis acids like H_3_PO_4_ or H_2_SO_4_ and bases like KOH, etc. can cause drastic changes in the porous texture of the carbon derived from banana empty fruit bunches, eucalyptus wood powder and mangosteen fruit shells [10,44,45].

#### 2.4.4. Thermogravimetric (TGA/DTG) Analysis

**Figure 8:**
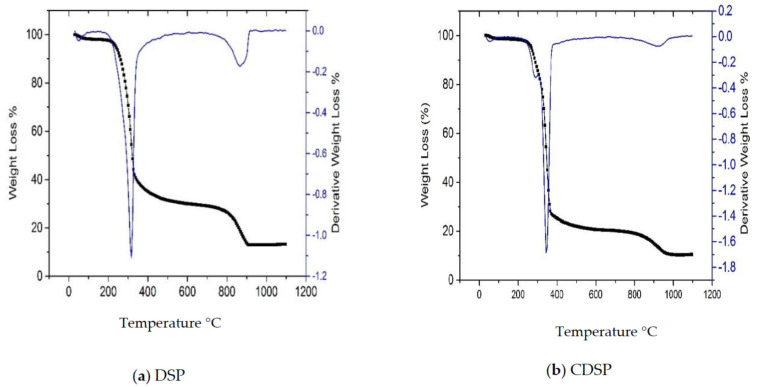
Thermogravimetric (TGA) and DTG profiles: (**a**) DSP and (**b**) CDSP samples.

The thermal stability profiles of DSP and CDSP were determined to observe the proportions of carbon, moisture, volatile materials and ash residues inside the samples. The thermal degradation curves for DSP and CDSP are plotted and shown by Figure 8a,b, respectively. Moisture was evaporated from both the samples at the first degradation phase at temperatures 70–120 °C. Hemicellulose and cellulose degradation took place for DSP around 200–400 °C [34]. Lignin decomposition took place from 200 to 800 °C [34]. Compared to the DSP sample, a higher carbon content was determined in the CDSP sample. The CDSP sample showed 1-higher thermal stability than the DSP sample. DTG_max_ for DSP and CDSP samples were 310.44 °C and 322.21 °C. Due to activation, the CDSP sample contained a larger amount of stable carbon content, leading to an enhanced thermal stability. Thermogravimetric Data is listed in Table 7.

#### 2.4.5. Energy-Dispersive X-ray (EDX) Analysis

EDX analysis was carried out for the DSP and CDSP samples to observe the proportion of carbon, oxygen and other mineral constituents. The results obtained after EDX analysis of the DSP and CDSP samples are shown by Table 8. Both the samples contained carbon and oxygen as the main constituents. The carbon content in the DSP sample was 43.11%, which was increased significantly after activation, around 86.76%. However, the oxygen content was more reduced in the CDSP sample than the DSP sample due to the volatilization of oxygen and hydrogen and decomposition of lignin during the activation process. Trace amounts of Zn were detected in the CDSP sample as the activation process was carried out in presence of Zn(CH_3_CO_2_)_2_·2H_2_O salt.

#### 2.4.6. Surface Functional Group Analysis

Surface functional groups onto the DSP and CDSP samples were analyzed using the FTIR technique and listed in Table 9. It is observed that, after activation, a lot of peaks were absent or shifted the frequency level. A similar phenomenon was reported for activated carbon obtained from pistachio shells [50,51]. A lot of oxygen-containing groups after activation disappeared due to aromatization of the carbon structure [10,52]. Some minor peaks around 500–800 cm^−1^ representing mainly aliphatic groups disappeared in the CDSP sample. The peak intensities around 3300–3500 cm^−1^ were reduced in the CDSP sample, reflecting the loss of moisture inside the sample after activation. It was reported earlier also that, after activation, mangosteen fruit shell and date pit-based activated carbon also lost some aliphatic groups present in this region (500–800 cm^−1^) [10,52]. The peak around 1000–1100 cm^−1^ reflecting ester or phenolic groups was present in both the raw DSP and CDSP samples. However, in the CDSP sample, the peak intensities reduced drastically. A similar trend was observed after the activation of rice straw using KOH [52].

## 3. Materials and Methods

### 3.1. Materials

The date seeds (DSP) were collected and washed several times to remove the mesocarps of fresh date fruits and were then oven-dried for 3 days at 90 °C. The dried seeds were ball-milled into powder, washed with hot deionized water and dried in a vacuum oven at 100 °C for more than five hours, until the weight of the powder was constant [32]. Six grams of date seed powder was mixed with 3 g of weak Lewis acid of zinc acetate dehydrate salt (Zn(CH_3_CO_2_)_2_·2H_2_O), where the ratio of seed powder to salt was kept constant around 2:1. The mixture was stirred for 3 h to ensure homogeneity. Afterward, the mixture was carbonized in the furnace (OTF-1200X MTI Corporation) under the flow of nitrogen gas (200 mL/min) to create an inert atmosphere. The temperature was increased slowly at the rate of 5 °C/min until it reached to 1000 °C and held at that temperature for 60 min. Temperature, time and gas flow rate were optimized during preliminary screening studies to ensure the maximum removal percentages of naphthalene (C_10_H_8_). The activated sample (CDSP) was withdrawn and washed vigorously with hot water and dried overnight at 60 °C under vacuum. The ratio between DSP powder and zinc acetate was kept at 2:1 to avoid the presence of significant amounts of zinc residuals inside the activated sample (CDSP).

Naphthalene (C_10_H_8_) and zinc acetate dehydrate salt (Zn(CH_3_CO_2_)_2_·2H_2_O) having purity of 99% were procured from Sigma Aldrich, USA. The stock solution was prepared having a concentration of 1000 ug/L. The PAHs used here as adsorbate naphthalene (C_10_H_8_) were further diluted to have the required concentration daily before the adsorption process.

### 3.2. Adsorption Experiments

The batch adsorption process was carried out to determine the adsorption isotherms and kinetics parameters. Adsorption of naphthalene (C_10_H_8,_) on DSP and CDSP was carried out at room temperature (30 °C). Different concentrations of C_10_H_8_ ranging from 12.5 ppb to 75 ppb were prepared, and 50 mL of each sample was transferred in 150-mL conical flask. Ten milligrams of adsorbate (DSP and CDSP) were mixed with each of the liquid samples (C_10_H_8_), having different concentrations prepared here. The liquid sample containing the adsorbent materials (CDSP and DSP) was stirred for a given time range (5–30 min) in a thermal water bath shaker to maintain a constant temperature of 30 °C at a pH of 6.5. Agitation speed was kept constant at 200 rpm during adsorption experiments. At regular intervals of time, the liquid samples were withdrawn, and the residual concentration of the adsorbate was analyzed using Equation (1) [10].

(1) $$q_{e\;}=\;\frac{\left(C_{0\;}-C_{e}\right)}{W}$$

Here, *C*_0_ and *C_e_* represent the initial and final equilibrium concentrations of naphthalene in the liquid phase, and *q_e_* (μg/g) shows the amount of naphthalene (C_10_H_8_) adsorbed over the surface of DSP and CDSP at the equilibrium condition.

Equilibrium data was analyzed using the linear equations of Langmuir, Freundlich and Temkin isotherm models. The rate of reaction or system kinetics with the mechanism was analyzed using the pseudo-first order, pseudo-second order and intraparticular diffusion models, respectively.

The nonlinear form of Langmuir Equation (2) can be linearized to give Equation (3) [10,53].

(2) $$q_{e}\;=\;\frac{K_{L}C_{e}}{1\;+\;a_{L}C_{e}}$$

(3) $$\frac{C_{e}}{q_{e}}\;=\;\frac{1}{q_{max}K_{L}}+\;\frac{1}{q_{max}}$$

Here, *q_m_* (mg/g) is the maximum monolayer adsorption capacity when the surface of the adsorbent is homogeneous, *K_L_* (L/mg) is the Langmuir constant, which corresponds with the binding energy of the sorption [10,53]. Based on the assumptions of the Langmuir model, the surface is homogeneous, having a fixed number of vacant sites. The vacant sites are similar in size and shape and can hold one molecule per vacant or sorption site, forming a monolayer over the adsorbent’s surface [10,53]. After the sorption of each molecule, an equivalent amount of heat energy can be released [10,53].

Heterogeneous surface sites where the surface is nonhomogeneous to adsorb a multilayer of adsorbate can be represented by following the nonlinear form of Freundlich Equation (4) [10,54]. The linear form of the Freundlich equation can be expressed by Equation (5) [54].

(4) $$q_{e\;}=\;K_{f}C_{e}^{1/n}$$

(5) $$Ln\;q_{e}=LnK_{f}+\;\frac{1}{n}Ln\;C_{e}$$

Here, *K_f_* represents the Freundlich constant (mg/g) (L/mg)^1/*n*^ and 1/*n* shows the affinity of the sorption [10,54]. According to the Freundlich isotherm, the sorption process linearly varies at lower pressure, but at higher pressure, it becomes independent [54].

According to the Temkin isotherm, the heat of the sorption reduces gradually with the degree of surface coverage and indirect adsorbate-adsorbent interactions take place during the adsorption process. The nonlinear and linear forms of the Temkin model are expressed by Equations (6) and (7) [10,55].

(6) $$q_{e}=\;\frac{RT}{b}\;LnK_{T}C_{e}$$

(7) $$q_{e}=\;\frac{RT}{b}Ln\;K_{T}+\;\frac{RT}{b}\;LnC_{e}$$

Here, *RT/b* = B is the Temkin constant, which corresponds with the heat of the adsorption, *K_T_* (L/g) is the binding constant, T °K is the absolute temperature of the solution and *R* = 8.314 J/mol.K) is the universal gas constant [55].

For the process scale-up, the equilibrium data was analyzed through pseudo-first and second order reaction kinetics using the following Equations of (8) and (9).

(8) $$\log\left(q_{e-\;}q_{t}\;\right)\;=logq_{e\;}-\frac{k_{1}}{2.303}\;$$

(9) $$\frac{t}{q_{t}}=\frac{1}{k_{2}q_{t}^{2}}\;+\;\frac{t}{q_{e}}$$

Here, $q_{t}\;\left(\mu g/g\right)$ and $q_{e\;}\left(\mu g/g\right)$ showed the adsorption quantity of naphthalene onto the surface of the adsorbent in any time *t* (minutes) and equilibrium contact time, respectively. $k_{1}\;\left(\min^{-1}\right)\;and\;k_{2}\;\left(\min^{-1}\right)$ are the first order and second order rate constants, and *t* (min) is the contact time for the sorption [10,34,35].

The equilibrium data was further fitted with the intraparticle diffusion model to analyze the mechanism of diffusion of the naphthalene molecule (C_10_H_8_) inside the pores of the DSP and CDSP adsorbents using the following Equation (10) [10,34,35]:

(10) $$q_{t\;}=k_{dif}\;t^{0.5}\;-C_{i}$$

Here, *K_dif_* (μg/g.h^0.5^) is the intraparticle diffusion rate constant, and *C_i_* is the boundary layer effect.

The Elovich equation was earlier used to observe the kinetics of the chemisorption of gases onto the solid surface. However, this kinetic equation can be also used to investigate the liquid phase sorption process [10]. The equilibrium adsorption data for DSP and CDSP was analyzed further using the Elovich equation using Equation (11) [10].

(11) $$qt=\;\frac{1}{b}\;Ln\left(ab\right)+\frac{1}{b}Lnt$$

Here, *a* (μg/g.h^−1^) represents the initial rate of adsorption, and *b* (mg/g) denotes the energy of the.

### 3.3. PAHs (C_10_H_8_) Analysis

The concentration of the PAH model compound (napththalene—C_10_H_8_) taken here as the adsorbate was analyzed using Gas chromatography–mass spectrometry–GC-MS/MS (GCMS TQ 8030, Shimadzu) following our previous method published elsewhere [31]. The column used was the Rxi-1 ms (Restek, Bellefonte, PA, USA), which has an internal diameter of 0.25 mm and film thickness of 0.25 µm. The injection temperature was 300 °C. The carrier gas used here was helium (99.999%), and its flow rate was fixed at 1.0 mL/min. The injection mode was changed to make the injection volume of 1.0 µL. The oven temperature was ramped from 40 °C to 250 °C at a rate of 10 °C/min without any hold, and then, another ramping was set from 250 °C to 300 °C at a rate of 5 °C/min with holding for 5 min. The gas flow rate inside the oven was set at 1.5 mL/min. The temperature of the detector was 230 °C.

### 3.4. Characterization of Date Seed (DSP) and Date Seed-Based Activated Carbon (CDSP)

The BET surface area and porosity of the adsorbents (DSP and CDSP) were analyzed using a Micrometrics Tristar II Surface Area Analyzer (USA). Before taking a N_2_ gas adsorption-desorption isotherm at 77 K, the CDSP sample was outgassed for 4 h at 300 °C under vacuum to remove any water content inside the sample. A BET curve was plotted until the relative pressure (P/P_0_) was 1 to calculate the surface area [33,34]. The BET equation and pore diameter, along with a *t*-plot analysis to observe the micropore mesopore distribution, was calculated using Micropore version 2.26 software, USA, available with the instrument [33,34]. The macropore volume of the activated samples (CDSP) was measured using mercury intrusion porosimetry using a Micrometrics IV 9500 Autopore porosimeter, UK, where the pressure was kept constant at 124 MPa, contact angle 130 °C and mercury surface tension was mNm^−^^1^. An ultimate analysis—that was C, H, N, O and others—in the DSP and CDSP samples were measured using PerkinElmer, Series II 2400, Japan. A Fourier-transform emission scanning electron microscope (FESEM, JEOL-JSM-6610LV) was used to observe the surface morphological feature of the DSP and CDSP. A thermogravimetric analysis was carried out using Mettler Toledo Star SW-901, Japan to determine the percentages of the moisture content, volatile materials, fixed carbon and ash residues in DSP and CDSP samples. For the thermogravimetric (TGA) analysis, 10 mg of each sample was heated until 1100 °C under a 5-mL/min nitrogen gas flow, and the heating rate was around 5 °C per minute. An energy-dispersive X-ray (EDX) analysis was carried out for the DSP and CDSP samples using a JEOL-JSM–6380 LA, UK. FTIR-NICOLET 6700 (Fourier-transform infrared spectroscopy) was used to observe functional groups over the CDSP and DSP samples.

## 4. Conclusions

In this research, date seeds (DSP) were used to prepare activated carbon (CDSP) using the one-step chemical activation method. The activation process used here did not use conventional harsh Lewis acids like H_2_SO_4_ or H_3_PO_4_ or a strong Lewis Base like NaOH, LiOH or KOH. The activation process was carried out using weak Lewis acids of zinc acetate dihydrate salt (Zn(CH_3_CO_2_)_2_·2H_2_O) in the presence of nitrogen gas. The surface area enhanced drastically after the chemical activation process using Zn(CH_3_CO_2_)_2_·2H_2_O. Based on the BET isotherm shape, it was concluded that the activated sample is mainly microporous in texture, with a certain proportion of mesopores. Due to the activating effect of weak Lewis acids of zinc acetate dihydrate salt (Zn(CH_3_CO_2_)_2_·2H_2_O) at higher temperatures, some micropore walls may disintegrate, leading to the formation of mesopores. The sorption performance of both the adsorbent (raw DSP and CDSP) materials was compared for the removal of naphthalene (C_10_H_8_) from synthetic wastewater. The prepared activated sample (CDSP) showed a higher sorption performance, rather than the DSP sample, for the removal of naphthalene (C_10_H_8_) from a synthetic aqueous solution. At room temperature (30 °C), the equilibrium isotherm data was fitted with linear regression equations of the Langmuir, Freundlich and Temkin isotherm models. A comparatively higher correlation coefficient, R^2^, was obtained for the Langmuir isotherm model for the adsorbate (C_10_H_8_) for adsorption onto the DSP and CDSP samples, which indicated homogeneous surface sites with monolayer sorption processes. The exponent, n, obtained from the Freundlich process was below 1 for adsorption onto the DSP and CDSP samples, indicating a favorable sorption process. The equilibrium data followed the pseudo-second order and Elovich kinetic models. This indicated that the chemisorption process took place for naphthalene (C_10_H_8_) onto the surfaces of the DSP and CDSP samples. The research findings indicated that the unactivated raw DSP and activated CDSP have great potential for the removal of a preselected poly aromatic hydrocarbon (naphthalene, C_10_H_8_) from a polluted aqueous stream.

## Figures and Tables

**Table 1:** Estimated values for constants from the Langmuir, Freundlich and Temkin models for the adsorption of naphthalene (C_10_H_8_) onto date seed powder (DSP) and carbon date seed powder (CDSP).

Sample	Langmuir		Freundlich			Temkin		
	*K_L_* (L/μg)	*R* ^2^	*K_F_* (μg/g) (L/μg)	*n*	*R* ^2^	*K_T_* (L/μg)	B	*R* ^2^
DSP	142.86	0.930	0.65	405.45	0.751	0.10	241.25	79.13
CDSP	10000	0.991	0.86	52.46	0.873	0.05	51.32	77.76

**Table 2:** The estimated values for the pseudo-first and second and intraparticle diffusion models for the adsorption of naphthalene (C_10_H_8_) onto DSP.

Pseudo-First Order Kinetics Model					Pseudo-Second Order Kinetic Model			Intraparticle Diffusion Model		
Initial Conc. (ppb)	Exp. *q_e_* (μg/g)	Cal. *q_e_* (μg/g)	*K*_1_ (Min^−1^)	*R* ^2^	Cal. *q_e_* (μg/g)	*K*_2_ (Min^−1^)	*R* ^2^	*K_dif_* (μg/g.h^0.5^)	*C_i_*	*R* ^2^
12.5	48.35	2.06	0.19	0.951	50.51	0.010	0.999	2.42	32.98	0.697
25	109.55	1.10	1.12	0.715	109.89	0.069	1.000	0.39	107.02	0.747
37.5	180.78	1.45	0.13	0.812	181.82	0.044	1.000	0.60	176.80	0.783
50	238.20	1.48	0.12	0.961	238.10	0.044	1.000	0.58	234.30	0.850
62.5	309.89	2.15	2.01	0.971	312.50	0.026	1.000	1.35	301.31	0.695
75	369.06	2.21	0.20	0.9996	384.62	0.008	1.000	4.10	363.42	0.572

**Table 3:** The estimated values for the pseudo-first and second and intraparticle diffusion models for the adsorption of naphthalene (C_10_H_8_) onto CDSP.

Pseudo-First Order Kinetics Model					Pseudo-Second Order Kinetic Model			Intraparticle Diffusion Model		
Initial Conc. (ppb)	Exp. *q_e_* (μg/g)	Cal. *q_e_* (μg/g)	*K*_1_ (Min^−1^)	*R* ^2^	Cal. *q_e_* (μg/g)	*K*_2_ (Min^−1^)	*R* ^2^	*K_dif_* (μg/g.h^0.5^)	*C_i_*	*R* ^2^
12.5	58.90	2.14	0.16	0.8372	63.69	0.003	0.995	4.56	29.90	0.691
25	123.7	1.07	0.06	0.231	123.46	0.047	1.00	0.63	119.65	0.726
37.5	190	1.63	0.06	0.559	192.31	0.021	1.00	1.16	182.40	0.805
50	247.89	1.41	0.07	0.468	250.00	0.023	1.00	1.61	237.73	0.475
62.5	304.90	1.78	0.12	0.431	312.50	0.005	0.999	1.69	262.090	0.767
75	379.64	1.51	0.08	0.406	384.62	0.012	1.000	1.69	368.07	0.767

**Table 4:** The estimated values for the Elovich equation model parameters for the adsorption of naphthalene (C_10_H_8_) onto DSP and CDSP.

DSP						CDSP				
Initial Conc. (ppb)	Exp. *q_e_* (μg/g)	Cal. *q_e_* (μg/g)	1/*b Ln*(*ab*)	1/*b*	*R* ^2^	Exp. *q_e_* (μg/g)	Cal. *q_e_* (μg/g)	1/*b Ln*(*ab*)	1/*b*	*R* ^2^
12.5	48.35	51.54	51.152	5.946	0.918	58.90	62.75	64.125	1.296	0.92
25	109.55	104.81	109.89	6.966	0.933	123.7	128.7	124.35	1.538	0.87
37.5	180.78	183.82	181.28	1.456	0.979	190	193.2	190.84	2.764	0.93
50	238.20	239.70	238.59	1.564	0.981	247.89	250.3	250.06	4.201	0.95
62.5	309.89	314.60	311.47	3.363	0.921	304.90	313.4	314.97	17.92	0.88
75	369.06	389.32	394.42	10.24	0.899	379.64	367.9	380.38	10.81	0.91

**Table 5:** Brunauer-Emmett-Teller -BET analysis of DSP and CDSP.

Sample	BET Surf. Area (m^2^/g)	*t*-Plot Micropore Area (m^2^/g)	*t*-Plot Ext. Surf Area (m^2^/g)	Mesopore Volume (cm^3^/g)	Micropore Volume (cm^3^/g)	Pore Diameter (nm)
DSP	7.3023	3.5021	4.8022	0.1141	0.0114	1.02
CDSP	725.8319	466.451	258.2031	0.4503	0.5823	3.96

**Table 6:** Ultimate analysis of DSP and CDSP.

Sample	Ultimate Analysis (wt%)						
	C%	H%	N%	O%	Others	H/C	O/C
DSP	41.93	6.01	0.86	49.98	1.22	0.143	1.19
CDSP	85.76	3.12	0.34	10.09	0.69	0.036	0.18

**Table 7:** Proximate analysis of the DSP and CDSP samples.

Element	DSP (%)	CDSP (%)
Moisture	6.55	1.22
Volatile Matter	41.77	5.03
Fixed Carbon	39.78	83.08
Ash Residues	11.90	10.67
DTG_max_	310.44 °C	322.21 °C

**Table 8:** Energy-dispersive X-ray (EDX) analysis of DSP and CDSP.

Element	DSP (%)	CDSP (%)
C	43.11	86.76
O	21.66	7.55
H	9.221	1.22
Mg	0.99	0.53
P	2.34	1.56
K	2.23	1.98
Cl	0.56	0.18
Ca	19.92	-
Zn	-	0.21

**Table 9:** Surface functional groups present on the DSP and CDSP samples.

	Frequency Level for Peaks		
No.	Raw DSP	CDSP	Peak Assignment
1	624.78	-	Bending Vibration of –C-O-H
2	856.78	-	-C-H Band for Out-of-Plane Bending vibration reflecting the presence of aromatic/benzene derivatives
3	1099.57	1100.78	-C-O-C Band Stretching showing ester linkages
4	1345.67	-	-CH_3_ Band
5	1578.87	1581.23	-COOH Functional Group
6	1634.77	-	-COOH Functional Group
7	1788.55	-	C∞O Band for Stretching Vibration
8	2367.66	-	C-H Bending Vibration
9	2989.33	-	-O-H Band for Stretching Vibration
10	3477.67	3388.90	-O-H Band for Stretching Vibration
